# Chronic Osteomyelitis - Bacterial Flora, Antibiotic Sensitivity and Treatment Challenges

**DOI:** 10.2174/1874325001812010153

**Published:** 2018-03-30

**Authors:** Kuzma Jerzy, Hombhanje Francis

**Affiliations:** 1 Faculty of Medicine and Health Sciences, Divine Word University (DWU) and Modilon General Hospital (MGH), Madang, Papua, New Guinea; 2 St. Mary's School of Nursing (DWU), Rabaul Campus P.O.Box 58, Kokopo East New Britain Province, PNG, Papua, New Guinea

**Keywords:** Antibiotic resistance, Debridement, Septic non-union, Topical antibiotics, Chronic osteomyelitis, One-stage treatment

## Abstract

**Background:**

Chronic osteomyelitis is a catastrophic sequel of delayed diagnosis of acute osteomyelitis.

**Objectives:**

The objectives of the study were to determine bacterial flora and antibiotic sensitivity, and to evaluate the outcome of an aggressive surgical approach to chronic osteomyelitis.

**Methods:**

This is a single surgeon, prospective cohort study on 30 consecutive patients with clinically and radiologically diagnosed chronic osteomyelitis presented to a hospital. We prospectively recorded demographic, clinical, radiological features, treatment protocol, microbiologic results of culture and sensitivity. The main treatment outcome measures were clinical signs of eradication of infection.

**Results:**

Microbiologic results showed that Gram-negative and mixed flora accounts for more than half of chronic osteomyelitis cases while *Staphylococcus aureus* was a dominating single pathogen (39%). We detected a high resistance rate to common antibiotics, *e.g.* 83% of *S. aureus* isolates were resistant to oxacillin (MRSA). The mean duration of bone infection was 4.2 years (3 months to 30 years) and the mean number of operations was 1.5 (1-5) . The mean follow-up was 15 months (12-18 months). Infection was eradicated in 95% (21 out of 22) treated by a single procedure and in all patients (n=8) by double procedure.

**Conclusion:**

Presented the high rate of MRSA strains is alarming and calls for updating of the antibiotic therapy guidelines in the country. Good results in treatment of chronic osteomyelitis can be achieved by a single-stage protocol including radical debridement combined with systemic and topical antibiotic.

## 1 INTRODUCTION

Chronic osteomyelitis is a catastrophic sequel of delay in diagnosis and management of the acute stage of the disease. It presents complex therapeutic challenges and imposes a high cost on healthcare provision. In contrast to developed countries, in low-resource countries, chronic osteomyelitis is much more common [1-6]. Inadequate health care setting frequently combined with patient malnutrition makes chronic osteomyelitis long lasting and causing more severe disability [3, 7, 8]. Treatment of chronic osteomyelitis, apart from prolonged antibiotic therapy requires radical debridement often resulting in bone and soft tissue defect which requires complex reconstructive procedures. In a low-resource setting not all reconstructive procedures can be implemented [1, 8].

Commonest pathogen responsible for chronic osteomyelitis remains *Staphylococcus aureus* [1, 6-9], while Methicillin-Resistant *S. Aureus* (MRSA) rates range from 10% to 59% [1, 3, 7-12]. Gram-negative and polymicrobial infections were commonly reported [1, 10, 13].

The primary aim of this study was to analyze bacterial flora and antibiotic sensitivity underlying chronic osteomyelitis. The secondary aim was to evaluate and compare the outcome of single- and double-stage clinical protocols for chronic osteomyelitis.

## 2 MATERIALS AND METHODS

### 2.1 Study Design

A single surgeon, prospective cohort study of 30 consecutive patients with chronic osteomyelitis treated in one institution was conducted between January 2014 and May 2016. The study was approved by the university (No UREC 2-2012) and hospital research ethics committees and all patients gave informed consent to participate in the study.

### 2.2 Inclusion and Exclusion Criteria

Patients with chronic osteomyelitis were included in the study. Chronic osteomyelitis was defined as presence for a minimum of 4 weeks clinical and radiological features of bone infections accompanied by at least one of the following: the presence of a sinus, a sequestrum, an intra-operative pus and positive microbiological cultures. Characteristic radiological features included the presence of bone destruction, sequestum and new bone formation. Informed consent was obtained from patients or guardians. Patients who had sub-clinical infection, shorter than 4-week history, refused informed consent or who withdrew their consent in the course of the study were excluded in the final analysis.

### 2.3 Sampling Procedure

Pus swab specimens were obtained deep from discharging sinuses or collected intraoperatively and placed into the Stuart Transport Medium and immediately transported to the laboratory. Care was taken to avoid the skin contamination. The specimens were Gram-stained and cultured on blood Agar Media and MacConkey Agar Media.

### 2.4 Microscopic Examination and Culture

To differentiate the organisms isolated, Gram-staining, colony characteristics were applied and the bacterial species, biochemical tests were performed. We used antibiotic-impregnated discs (Oxoid, Scoresby, Australia) to test antibiotic sensitivity of the isolates. Specimens were classified according to the Clinical and Laboratory Standards Institute Guidelines Institute [14] as sensitive, intermediate or resistant (Table **2**).

### 2.5 Data Collection

We prospectively recorded on standard research forms demographic and clinical features (Table **1**). The nutrition status for children was measured as percentage of desired body weight for age (normal 80-100%, underweight 60-79%, severe malnutrition as less than 60%).The Cierny-Mader anatomic (determined at operation) and host status classification was applied. Radiological and microbiological outcomes were confirmed by assessors who were not involved in the management of the patients.

### 2.6 Surgical Management

Patients were managed according to single- or double-stage clinical protocols for chronic osteomyelitis. The single-stage involved excision of sinuses, metal removal, radical bone debridement aiming at removing all necrotic bone down to bleeding bone (‘paprika sign’), bone marrow cavity reaming where required. We paid special attention to the removal of all the biofilm by curettage, scrubbing and irrigation of the wound with saline solution. Post debridement, all patients had topical antibiotic application (gentamycin and rifampicin) and systemic antibiotic based on the previous culture and sensitivity test. The bone defect was filled with autogenic bone graft and/or calcium sulfate. In case of soft tissue defect, an attempt was made to cover the bone with well-vascularized muscles or fascio-cutaneous local flap.

For more complex cases with difficult soft tissue management requiring a local flap cover and/or significant bone defect (more than 1cm) requiring autogenous bone grafting we adopted a double-stage protocol. The first stage consists of radical debridement, removal of metal, wash out, topical antibiotics, covering stripped bone by muscle and a loose skin approximation. Usually, the second stage was performed after a week and involved repeated debridement, filling of bony defect with autogenous bone graft mixed with calcium sulfate and topical antibiotics, bone stabilization and soft tissue cover. Depending on the case we selected either external fixator in the first or internal fixation applied in the second stage.

### 2.7 Antibiotic Management

Most of our patients were on antibiotics on admission. While we stopped the antibiotic administration before the procedure, the period of 2 weeks without antibiotics for culture taking could not be followed. On the day of the procedure, we applied antibiotics based on the susceptibility results and continued intravenously for a week. Then, oral antibiotic therapy consisting of two antibiotics continued routinely up to 6 weeks.

### 2.8 Outcome Parameters

The main outcome of the combined treatment was healing of the sinuses and wounds, and lack of the infection recurrence. Treatment failure was defined as signs of recurrent infection proved by positive culture, sinus formation, pathological fracture and further surgery for bone infection. Secondary outcomes were the length of hospitalization and in case of septic non-union the time to bone union. Typically, the patients were followed up in 2, 6 and 12 weeks, 6 and 12 months; minimal follow up was set as 12 months.

### 2.9 Data Analysis

The descriptive data analysis was performed using the computer software SPSS version 15. Numerical data were expressed as arithmetic mean with standard deviation (mean ±SD), and categorical data as percentage representation.

## 3 RESULTS

### 3.1 Demographic Characteristics

Table **1** presents demographic and some clinical characteristics of the participants.

In 73% of the cases of the chronic osteomyelitis was caused by inadequate treatment of open fractures. Children less than 16 years old constitute 63% of the whole group. Antibiotic treatment prior to the admission could be one of the factors contributing to a relatively low white cells count for the participants.

### 3.2 Bacterial Profile

**Fig. (1):**
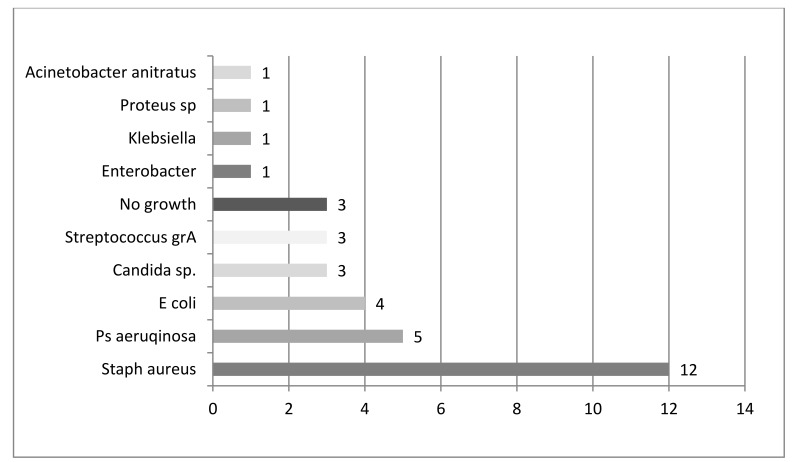
Bacterial profile of the patients with chronic osteomyelitis, tertiary hospital, PNG, 2014-2016. Legend: sp - species, grA - group A, Staph - *Staphylococcus*

The most common single pathogen was *S. aureus* (12 out of 31 positive cultures, 39%). Of 31 positive cultures Gram-negative bacteria accounted for 41% (n=13) and polymicrobial infection for 13% (n=4), and *Candida sp.* for 10% (n=3) (Fig. **1**). Microbiologic results showed that Gram-negative and mixed flora accounts for more than half (54%) of chronic osteomyelitis.

### 3.3 Antibiotic Sensitivity

The most alarming finding was that 83% of *S. aureus* strains were resistant to methicillin (MRSA) (Table **1**). In addition, almost two-thirds of *S. aureus* strains (67%) were also resistant to ceftazidime. We reported a high sensitivity of *S. aureus* to ciprofloxacin and erythromycin (100%), gentamycin (92%) and chloramphenicol (83%). Gram-negative bacteria showed a high sensitivity to ciprofloxacin (100%), ceftazidime (92%), gentamycin (75%) and chloramphenicol (60%) but lack of sensitivity to trimethoprim-sulfamethoxazole (0%) or low sensitivity to ampicillin (8%) and tetracycline (25%). Of note was detection of Gram-negative bacteria resistant to multiple drugs, *e.g.* *E. coli* sensitive only to ceftazidime or *Klebsiella pneumonie* and *Pseudomonas aeruquinosa* sensitive only to ceftazidime and ciprofloxacin.

### 3.4 Treatment Outcome

The mean duration of bone infection was 4.2 years (3 months to 30 years) and the mean number of operations was 1.5 (1-5). The mean hospital stay was 5.3 (range, 2-36) weeks. The mean follow-up was 15 months (12-18 months). Two patients out of 30 (7%) were lost to follow up; 1 from each treatment group. We applied an intention-to-treat principle and the missing observations were replaced by the median for the particular treatment group. Infection was eradicated in 95% (21 out of 22) treated by a single-procedure and in all (8) the double-procedure group. Two local recurrences occurred within 3 months, one was treated with additional procedures and one refused further surgery. Overall, adverse events were prolonged serous wound leakage in 3 cases (10%), 2 partial soleus muscle flap necrosis that healed after the debridement and skin grafting, 3 half pin infections and 2 delayed unions.

The management was tailored to patient’s characteristic. Eight children with chronic osteomyelitis of tibia (Cierny-Mader stage IV^A^) were treated by radical debridement in the form of the tibia de-roofing, topical and systemic antibiotics as well as covering of the debrided tibia by well vascularized muscle. There was one recurrence of infection (out of 8, 13%) in this subgroup.

Four patients with chronic infection and bone union with the presence of an intramedullary implant (Cierny-Mader stage IV^A^) were managed by implant removal, sinuses excision, bone canal reaming, washing, topical and systemic antibiotics. There was no recurrence of infection in this subgroup.

**Fig. (2):**
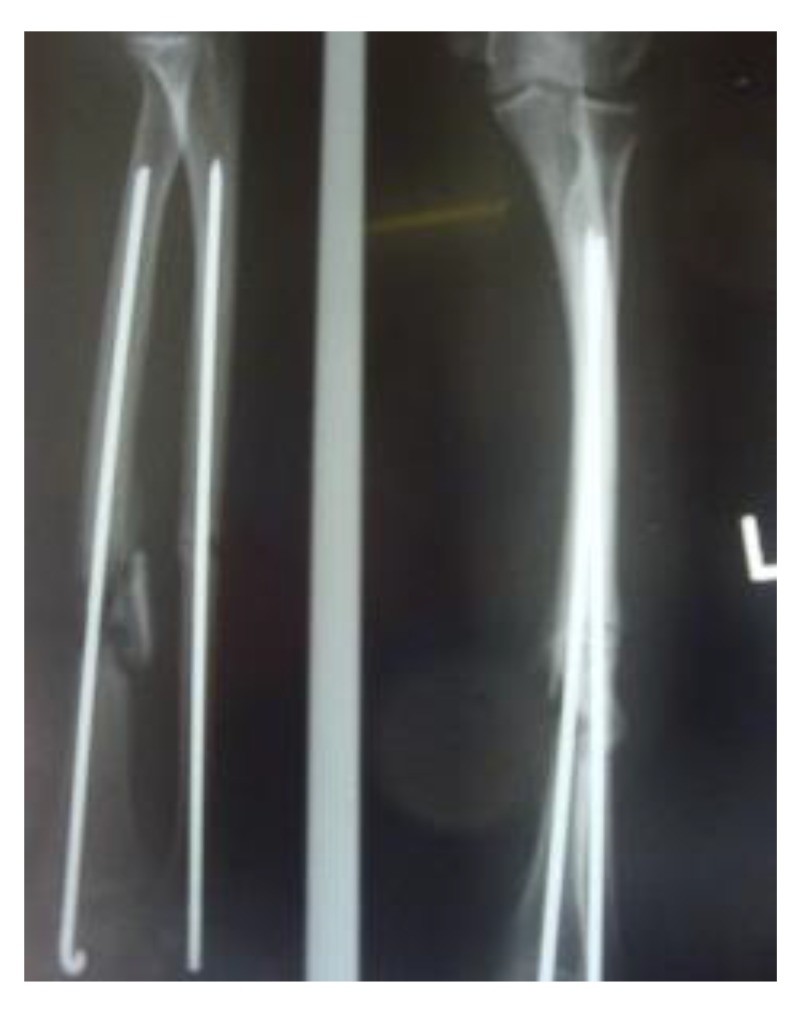
Case 1: A 40-year old female 5 months after Rush pin fixation, non-union of ulna and septic nonunion of radius with sequestrum, 2.2cm bone defect and sinus.

**Fig. (3):**
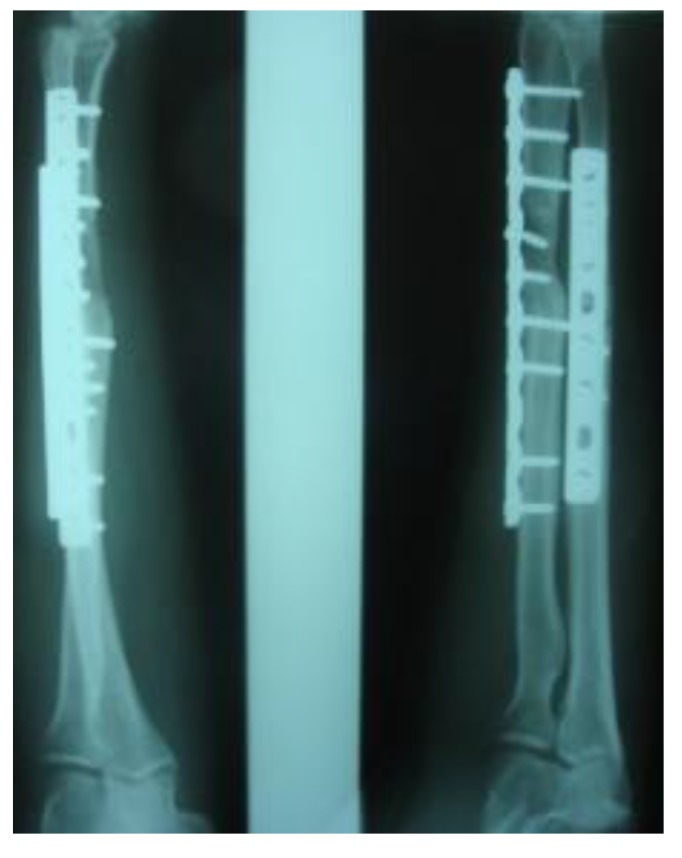
Case 1: Five months after two-stage approach: radical debridement with topical and systemic antibiotics followed in a week by a tri-cortical iliac bone graft and plating.

Three patients diagnosed as septic non-union with bone defect in ulna after Rusch pin stabilization were treated by a two-stage approach. The average length of bone defect was 2.3cm (1.5-3.2 cm). In the second stage, we applied tri-cortical iliac crest autologous bone graft impregnated with a topical antibiotic and DCP long plate, bone covering with muscle and suture. In all cases, eradication of infection and graft incorporation with bone union was achieved (Figs. **2** and **3**).

Septic non-union of humerus (n=3) were managed by one-stage protocol and a monoplane external fixator with compression. All healed with average union time of 3.9 (range 2.5 - 4.2) months without recurrence of infection. One patient sustained the radial nerve palsy which resolved.

**Fig. (4):**
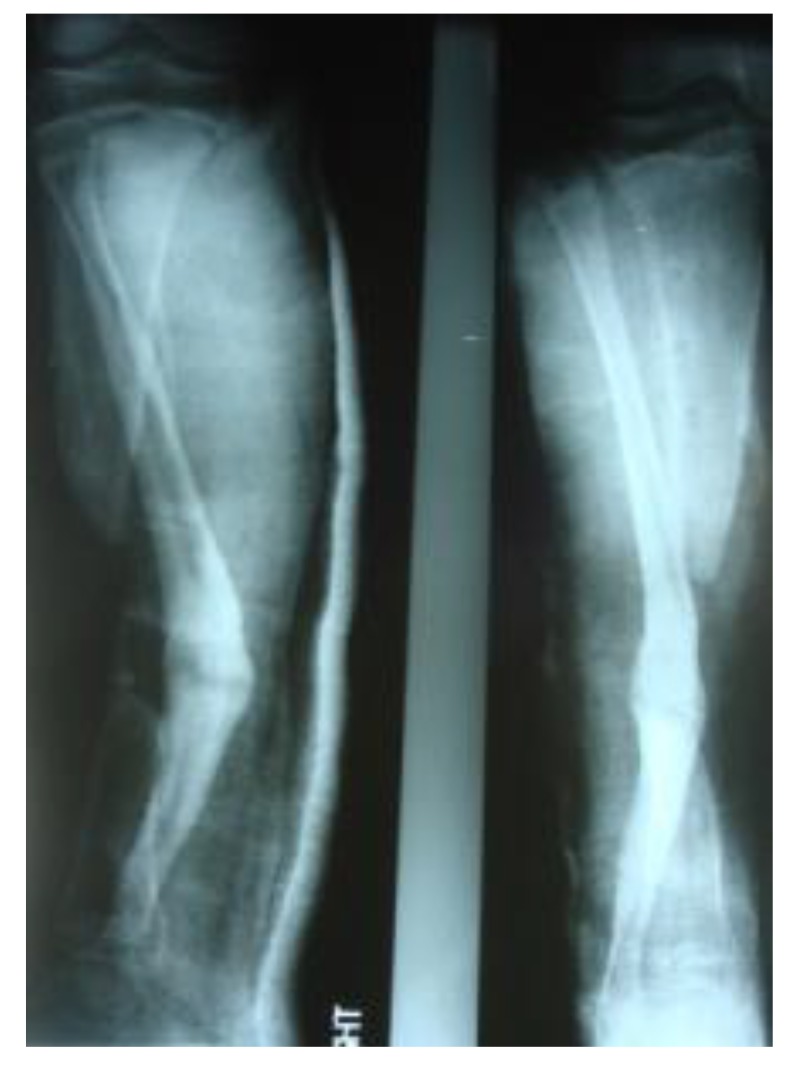
A 15-year-old boy 15 months post a car accident with chronic osteomyelitis and tibia defect of 7cm, inadequate soft tissue coverage (anterior tibia covered with split skin graft) and multiple sinuses. First stage included radical debridement, spacer from hydroxyapatite, topical antibiotics (rifampicin and gentamycin) and soleus flap cover, immobilization with a long slab.

**Fig. (5):**
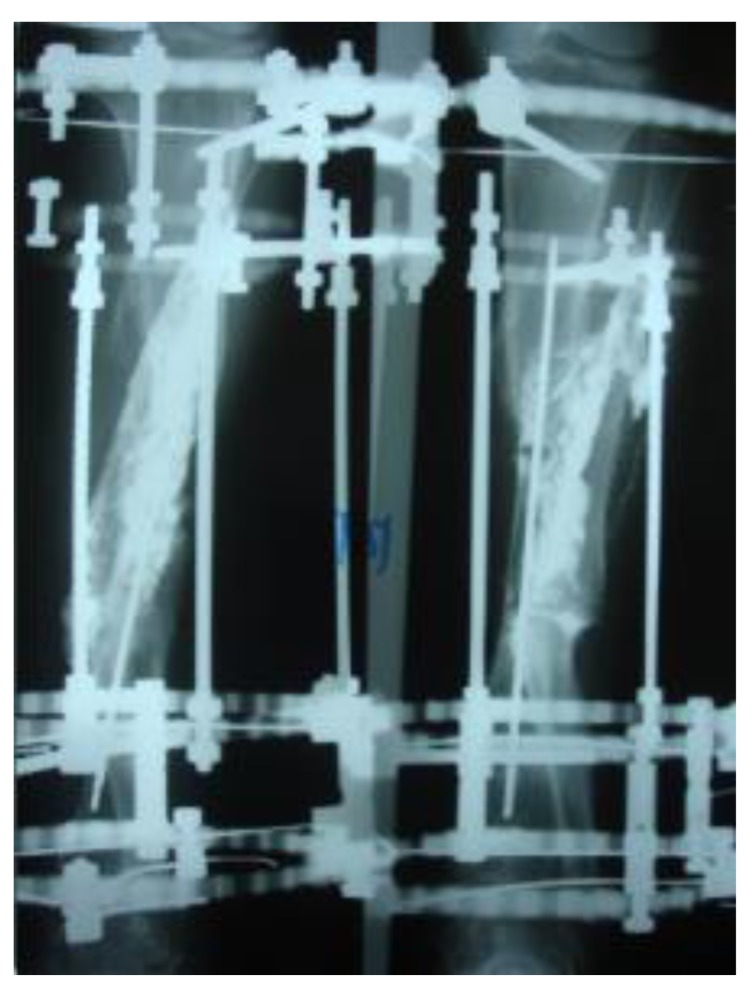
Case 2: Five months post 2^nd^ operation comprising contralateral free fibular graft stabilized by intramedullary rod, iliac crest graft, calcium sulfate with topical antibiotics and Ilizarov fixator. Lack of callus and gap at the proximal end of the fibular graft, injected 30ml of bone marrow with calcium sulfate, applied 4mm compression in 1mm steps daily.

The patients with septic nonunion of tibia (n=10) required in average 2.2 (range 1-5) procedures. In cases where well vascularized soft tissue cover could be assured and bone defect less than 1 cm we performed single-stage management. When tibia defect was larger than 1cm (average gap of 3 cm; 2.2 to 7cm) we adopted a two-stage protocol. In tibia septic non-union the bone was stabilized with the cast (n=2), locked intramedullary nail (n=1), Ilizarov external fixator with compression (n=6) and locking plate (n=1). In all cases but one fibular osteotomy was performed at different level than tibia fracture and more proximally than 6 cm from the ankle. The average union time confirmed on X-ray was 6.3 (range 6-10) months. For six patients with a larger bone defect, Ilizarov frame was applied. However, only 2 united without additional procedures in 3 months. After the external fixator removal, we applied the patellar tendon bearing cast with partial weight bearing until solid callus was shown on X-ray. For the patient with 7 cm-long tibia defect (Fig. **3**), we adopted induced membrane method (waiting time 5 weeks) then the contralateral free fibula graft was placed, stabilized with intramedullary rod and augmented by the iliac crest graft and calcium sulfate (Fig. **4**). Because of delayed union at the proximal end at 6 months, the bone marrow with calcium sulfate was injected into the non-union site and in 9 months the union was confirmed (Fig. **5**) and patient ambulating without pain.

**Fig. (6):**
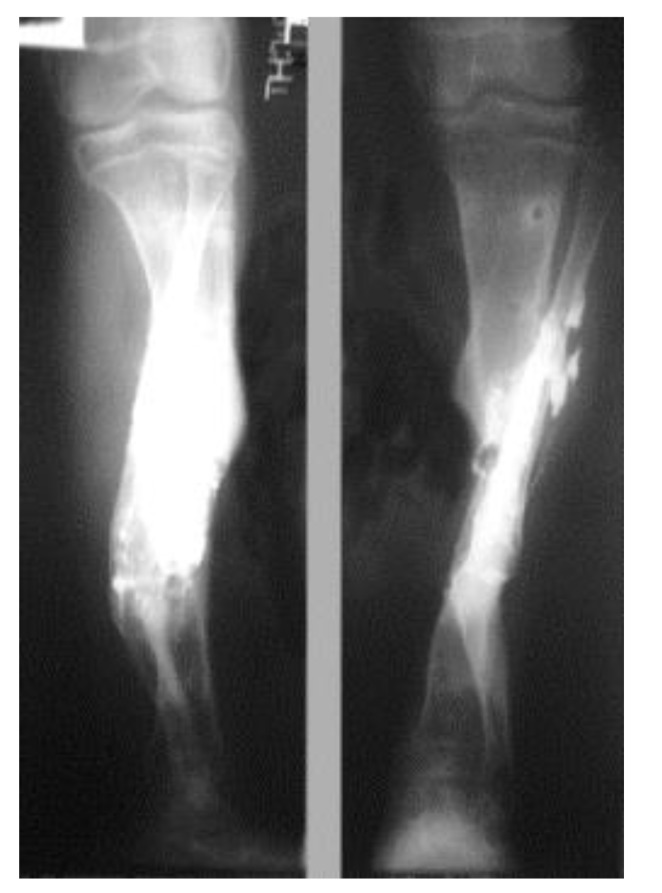
After 9 months from the bone grafting and Ilizarov application tibia union was achieved and patient was able to ambulate without pain, the limb was shortened 2 cm.

In 6 ring fixators we had 2 pin-site infections (33%), one superficial that responded to antibiotics and one required the pin removal. In 2 cases of chronic osteomyelitis of calcaneus (Cierny Mader stage III ^A^& III^BC^) with a soft tissue defect we applied two-stage approach achieving healing (Fig. **6**).

## 4 DISCUSSION

Despite progress in understanding of chronic osteomyelitis and advances in surgical techniques, chronic osteomyelitis still possesses treatment challenges.

### 4.1 Bacterial Flora and Susceptibility Pattern in Chronic Osteomyelitis

Our findings correspond to other authors that Gram-negative and polymicrobial flora are dominating pathogens responsible for chronic osteomyelitis [1, 10, 13] while *S. aureus* remains the single main pathogen responsible for osteomyelitis [1, 6-8, 12]. Alarming findings in our report was a higher rate (83%) of methicillin resistant *S. aureus* strains (MRSA) compared to other reports (10-59%) [1, 3, 6-9]. Common resistance to antibiotics among Gram-negative bacteria both in our series and other reports [1, 10, 13] could be possibly explained by the fact that many of them had undergone prolonged antibiotic therapy. Also, very low metabolisms in deeper layers of the biofilm are believed to predispose to development of strains resistant to antibiotics [15, 16].

### 4.2 Treatment of Chronic Osteomyelitis

In our study, we achieved a long-lasting suppression of infection in 97% of cases. However, what apparently looks like an excellent infection eradication rate might worsen in time as relapses of infection in chronic osteomyelitis have been recorded many years later [17, 18]. Review by Walter **et al*.* [16] on the management of chronic osteomyelitis showed that radical debridement combined with anti-bacterial chemotherapy leads to long-lasting suppression of infection in 70% to 90% of cases. Slightly higher rates of long-lasting infection suppression (88-95%) were reported by studies which added topical antibiotics to radical debridement with and systemic antibiotics [16, 19, 20]. Also, high rates of infection eradication have been reported by using various carriers for topical antibiotics such as impregnated collagen [21], calcium sulfate [20, 22-25], calcium sulfate with autogenous bone graft [26]. It is suggested that relatively rapid dissolution of antibiotics from bio absorbable material avoids prolonged low-level antibiotic release as in the case of impregnated PMMA beds and is believed to reduce the risk of growing antibiotic resistance [27]. The recurrence infection rate after PMMA carriers impregnated with antibiotics was reported to be above 10% [28, 29] and sometimes as high as 45% [30]. Promising effects in treatment of chronic osteomyelitis both in animal models and human studies were achieved by prolonged delivery of local antibiotic through the application of novel carrier systems such as microporous hydroxyapatite [31], biodegradable composite bone cement [32] and bioactive silicate glass scaffolds [33]. This type of delivery system offers longer and higher concentration of antibiotics at the infection site with diminished likelihood of toxicity [34].

We followed the conventional recommendation [1, 35] of a minimum 1 week of parenteral and continued up to 6 weeks with oral antibiotics. Although in chronic osteomyelitis some authors postulated longer periods of antibiotic therapy [36], it has been shown that post-debridement antibiotic parenteral treatment longer than one week and continuation of oral therapy beyond six weeks have not improved the outcome [37, 38].

There is now increasing interest in a single-stage protocol which reduces hospital stay. We achieved a high infection eradication rate following a single-stage protocol. Current treatment strategy in chronic osteomyelitis recognizes single- [20, 36] or double-stage clinical protocols [1, 12, 29]. Recent report on multiple debridement in conjunction with negative pressure wound therapy showed that increasing the number of revision procedures has not improved the rate of resolution of osteomyelitis [39].

Similar to others [1, 6, 12, 29, 35], we aimed the debridement at removing all necrotic bone down to bleeding bone (‘paprika sign’). The importance of radical debridement was demonstrated by Simpson **et al*.* [40] who reported that resection margin of 5 mm had no recurrence of infection; the smaller marginal resection group had 28% of infection recurrence rate while after simple debulking all patients had recurrence within 1 year.

To deal with bone defect, in our series, we utilized the following techniques: autogenous bone grafting mixed with calcium sulfate impregnated with antibiotics, free contralateral fibula graft and induced membrane technique.

Although McKee *et al* [22], after using calcium sulfate showed healing of 14 out of 16 non-unions in chronic-osteomyelitis, however, 9 of them received also autologous iliac crest bone grafts. Likewise, Borrelli *et al* [26]. have shown that adding calcium sulfate with antibiotic to autogenous bone graft improved treatment results in bone defect and nonunion in chronic osteomyelitis. Calcium sulfate materials showed effectiveness in eradication of infection but it was not reliable to fill the bone defect leading to pathological fractures in 5-8% of patients [20, 41].

In our study, 9 patients required different plastic-reconstructive procedures to cover the soft tissue defect. To effectively treat and prevent chronic osteomyelitis an orthopedic surgeon working in a low-resource environment should attain reconstructive plastic surgery skills. Apart from antibiotics and debridement, soft tissue coverage, and bone vascularity are important for long-lasting remission [1, 42].

### 4.3 Limitations of the Study

This study has several limitations. Firstly, due to a relatively small sample size, heterogeneity of cases and single hospital location, generalization of the results should be made with caution. Secondly, blood cultures may have strengthened our findings, but were not feasible and prior administration of antibiotics would have provided a low return. Further, not all specimens were taken as intraoperative biopsy; some were taken from sinuses discharge. It is argued that specimen taken from the sinuses could be contaminated by extraneous organisms from the superficial part [43]. Thirdly, this study is limited by the relatively short follow up (minimum 12 months), however, most recurrences present within one year of treatment [20]. Longer follow up could change a high infection eradication rate because of known cases of infection recurrence after more than one year [6, 13, 20, 29].

## CONCLUSION

The presented antibiotic resistance pattern in chronic osteomyelitis with the very high rate of MRSA strains is alarming and calls for updating of the antibiotic therapy guidelines in the country. Although treatment of chronic osteomyelitis is challenging, good results can be achieved by a single-stage protocol including radical debridement in conjunction with systemic and topical antibiotic. The knowledge of different reconstructive techniques is necessary to reconstruct osseous and/or soft tissue defects.

## Figures and Tables

**Table 1:** Demographic features of the patients with primary surgical infections admitted to tertiary hospital, PNG, 2014-2016.

**Variable**		**x̅(SD) or number (n)**	**Percentage**
Age		17.7(±12.3)	-
Gender	Male Female	n = 26 n = 4	87% 13%
Hemoglobin		11.5(±3.1)	-
White cells count		7(±2.5)	-
Nutrition	Normal Underweight Severe malnutrition	n = 20 n = 10 n = 0	67% 33% 0%
Location	Tibia Forearm Humerus Calcaneus Femur	n = 19 n = 5 n = 3 n = 2 n = 1	63% 17% 10% 7% 3%
C-M classification	III^A^ III^BC^ ***Total III*** IV^A^ IV^BL^ ***Total IV***	n = 13 n = 1 n** = 14** n = 8 n = 8 **n = 16**	**47%** **53%**
Smoking	Yes No	n = 5 n = 25	17% 83%
Pre - sample antibiotic	flucloxacillin chloramphenicol gentamycin	n = 10 n = 22 n = 30	-

Legend: x̅ - arithmetic mean, SD - standard deviation, n - number, C-M - Cierny-Mader classification of chronic, osteomyelitis, A - Host: Good immune system & delivery, BL - locally compromised host, BC - combined locally & systematically compromised host

**Table 2:** Summary of antibiotic sensitivity tests of bacteria obtained from patients with chronic osteomyelitis in tertiary hospital, PNG, 2014-2016.

**Micro-Organism**	**Antibiotic sensitivity in percentage (sensitive number/total tested number)**										
	Penc	Amp	CMP	Te	Gn	SXT	Caz	Cip	Ox	VA	Ery
*S. aureus*			83% 10/12		92% 11/12	42% 5/12	33% 4/12	100% 12/12	17**%** 2/12	100% 12/12	100% 12/12
*Streptococcus gr A*	100% 3/3		100% 3/3	100% 3/3		67% 2/3			100% 3/3		100% 3/3
Total gram-negative		8% 1/12	60% 6/10	25% 3/12	75% 9/12	0% 0/12	92% 11/12	100% 12/12			

Legend: Penc - penicillin, Amp - ampicillin, CMP - chloramphenicol, Te - tetracycline, Gn - gentamycin, SXT - trimethoprim-sulfamethoxazole, Caz - ceftazidime, Cip - ciprofloxacin, Ox - oxacillin, VA - vancomycin, Ery - erythromycin, grA - group A
